# Change in subfoveal choroidal thickness in diabetes and in various grades of diabetic retinopathy

**DOI:** 10.1186/s40942-018-0136-9

**Published:** 2018-09-12

**Authors:** Vikas Ambiya, Ashok Kumar, V. K. Baranwal, Gaurav Kapoor, Amit Arora, Nidhi Kalra, Jyoti Sharma

**Affiliations:** Base Hospital Delhi Cantonment, New Delhi, 110010 India

**Keywords:** Choroidal thickness, Subfoveal choroidal thickness, Diabetes mellitus, Diabetic retinopathy

## Abstract

**Background:**

To evaluate subfoveal choroidal thickness (SFCT) change in diabetes and in various grades of diabetic retinopathy (DR) in comparison to age-matched healthy subjects.

**Methods:**

This prospective observational study included 100 eyes of diabetic patients without DR (group D), 100 eyes with DR (group R), and 100 eyes of healthy subjects (group N). The assessment included demographics, duration of diabetes, comprehensive ocular examination, fundus photography with/without fundus fluorescein angiography, spectral domain optical coherence tomography with enhanced depth imaging to assess SFCT.

**Results:**

The SFCT was comparable between groups N (310.65 ± 37.34 µm) and D (308.48 ± 30.06 µm; P = 0.60), but was significantly lower in R (296.52 ± 21.41 µm; P < 0.01). The SFCT was significantly lower in proliferative DR (n = 36; SFCT = 284.56 ± 21.09 µm) as compared to non-proliferative DR (n = 64; SFCT = 303.25 ± 18.59 µm; P < 0.001). The SFCT had moderately negative correlation with severity of DR (R = − 0.50; P < 0.01). The difference in SFCT when compared with normal subjects was significant only in severe/very severe non-proliferative DR (294.47 ± 15.65 µm; P < 0.01) and in proliferative DR (284.56 ± 21.09 µm; P < 0.01). There was a negative correlation of SFCT with the duration of diabetes (R = − 0.41; P < 0.01).

**Conclusion:**

SFCT decreases with increasing duration of diabetes. The decrease is significant after the onset of severe DR, and is proportionate to the severity of DR.

## Background

The choroid is the only source of oxygen and nutrients to the outer retina and the retinal pigment epithelium. Although diabetic retinopathy (DR) is described as a disease primarily affecting the retinal microvasculature, the concept of diabetic choroidopathy came into light when it was first reported that there is a significantly higher loss of choriocapillaris in diabetic subjects than in aged control subjects [1]. The loss of outer retinal oxygenation may be the reason for the loss of cones in diabetic retina even when there is no retinopathy present [2, 3]. Doppler flowmetry has also shown decreased choroidal perfusion in early DR [4].

Studies on relationship of choroidal thickness (CT) with diabetes or with progression of DR have shown conflicting results, with some studies showing increase in thickness [5, 6] while others showing decrease in thickness of choroid [7–11] in diabetic patients or with increase in severity of DR. In view of paucity of definitive evidence of change in CT in association with diabetes/DR, we conducted a prospective observational study to compare the subfoveal choroidal thickness (SFCT) in: (1) diabetic patients with no evidence of DR; (2) in diabetic patients with DR; and to compare both with (3) age matched control subjects with no diabetes. We also aimed at comparing the difference in SFCT with increasing severity of DR.

## Methods

A prospective observational study was performed at a tertiary care eye institute from Jan 2018 to May 2018. This study was conducted after obtaining approval from the institutional ethical committee, and in accordance with the tenets of the Declaration of Helsinki. An informed consent was obtained from each study subject.

The inclusion criteria were: (1) age ≥ 18 years; (2) group D: eyes of known diabetic patients with no DR; group R: eyes of known diabetic patients with DR of any severity; group N: eyes of age matched control cases with no diabetes.

The exclusion criteria were: (1) Pan retinal laser photocoagulation ((PRP)/focal laser in the past 6 months; (2) history of vitreoretinal surgery; (3) vitreoretinal disorders other than diabetic retinopathy currently or in the past; (4) spherical equivalent of refractive error ≥ ± 6 D; (5) cataract surgery in the past 6 months; (6) any media opacity likely to cause attenuation of signal strength in OCT; (7) signal strength < 6/10 in OCT; (8) current or past history of accelerated hypertension; (9) pregnancy; (10) currently on drugs likely to alter the CT.

We defined diabetes according to World Health Organization guidelines as fasting plasma glucose ≥ 126 mg/dl or 2-h plasma glucose ≥ 200 mg/dl or being on antidiabetic medication. The controls (group N) had normal glycaemic values [12].

A detailed systemic and ocular history (onset of symptoms, present and previous treatment), the demography (age, gender), laterality, systemic comorbidities (diabetes and hypertension) were recorded. A detailed history of the duration of diabetes and glycaemic control was taken.

The clinical examination included assessment of the best corrected visual acuity in Snellen, spherical equivalent of the refractive status of the eye, slit lamp biomicroscopy with a contact lens or non-contact lens, indirect ophthalmoscopy, and digital fundus photography. DR was graded into mild (subgroup R1), moderate (subgroup R2), and severe/very severe (subgroup R3) non-proliferative DR (NPDR) or proliferative diabetic retinopathy (PDR; subgroup R4) as per ETDRS classification [13]. Digital fundus fluorescein angiography (FFA) was done at the discretion of the treating ophthalmologist if required for the staging of DR or for treatment. All eyes underwent optical coherence tomography (OCT) with enhanced depth imaging (EDI) to assess the SFCT, using spectral domain OCT (Carl Zeiss Meditec, Inc., 5160 Hacienda Drive, Dublin, CA 94568 USA). The assessment was made by a masked observer who was not aware of the diabetic status of the subjects. The subfoveal CT was measured using the EDI-OCT technique with two perpendicular foveal scans. The fovea was scanned with HD Cross 10-line protocol, with 5 horizontal and 5 vertical (8 times averaged) 6 mm B-scans, spaced 0.075 mm apart. The horizontal section and the vertical section, passing through the foveola, were selected. The vertical distance between the hyperreflective line of Bruch’s membrane and the innermost hyperreflective line of the chorio-scleral interface was taken in the two selected scans, and the average of the two scans (vertical and horizontal) was considered as the subfoveal CT. The various clinical and tomographic features were compared between the three groups followed by similar comparison among subgroups with different severities of DR.

## Statistical analysis

The numerical variables between any two groups were compared using Mann–Whitney U Test. The difference between proportions was analysed using Z-test. The correlation between severity of diabetic retinopathy and the CT was calculated using Pearson Correlation Coefficient. P-value of < 0.05 was considered as statistically significant.

## Results

The study included 100 eyes of 53 patients in group D (diabetic patients with no DR), 100 eyes of 53 cases in group R (diabetic patients with DR), and 100 eyes of 50 age matched normal subjects who were non-diabetic (group N).

**Fig. 1:**
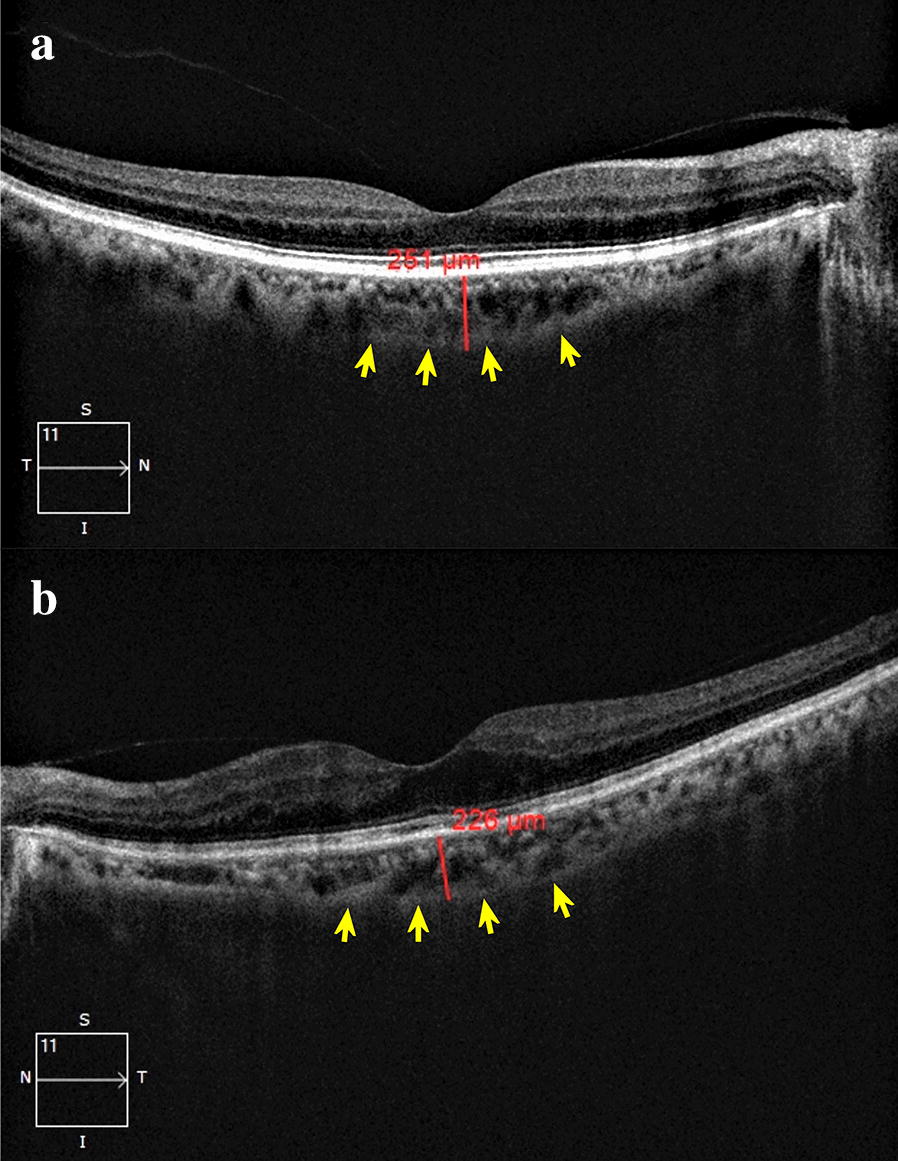
**a** Enhanced depth imaging-optical coherence tomography (EDI-OCT) image of the right eye of a 64 years old male subject with no diabetes, showing a well delineated layer of choroid with a clear demarcation of its outer limit (arrows), and a subfoveal choroidal thickness of 251 µm. **b** EDI-OCT image of the left eye of a 66 years old male diabetic subject with severe non-proliferative diabetic retinopathy, having a comparatively lower subfoveal choroidal thickness of 226 µm (outer limit of choroid labelled with arrows)

**Fig. 2:**
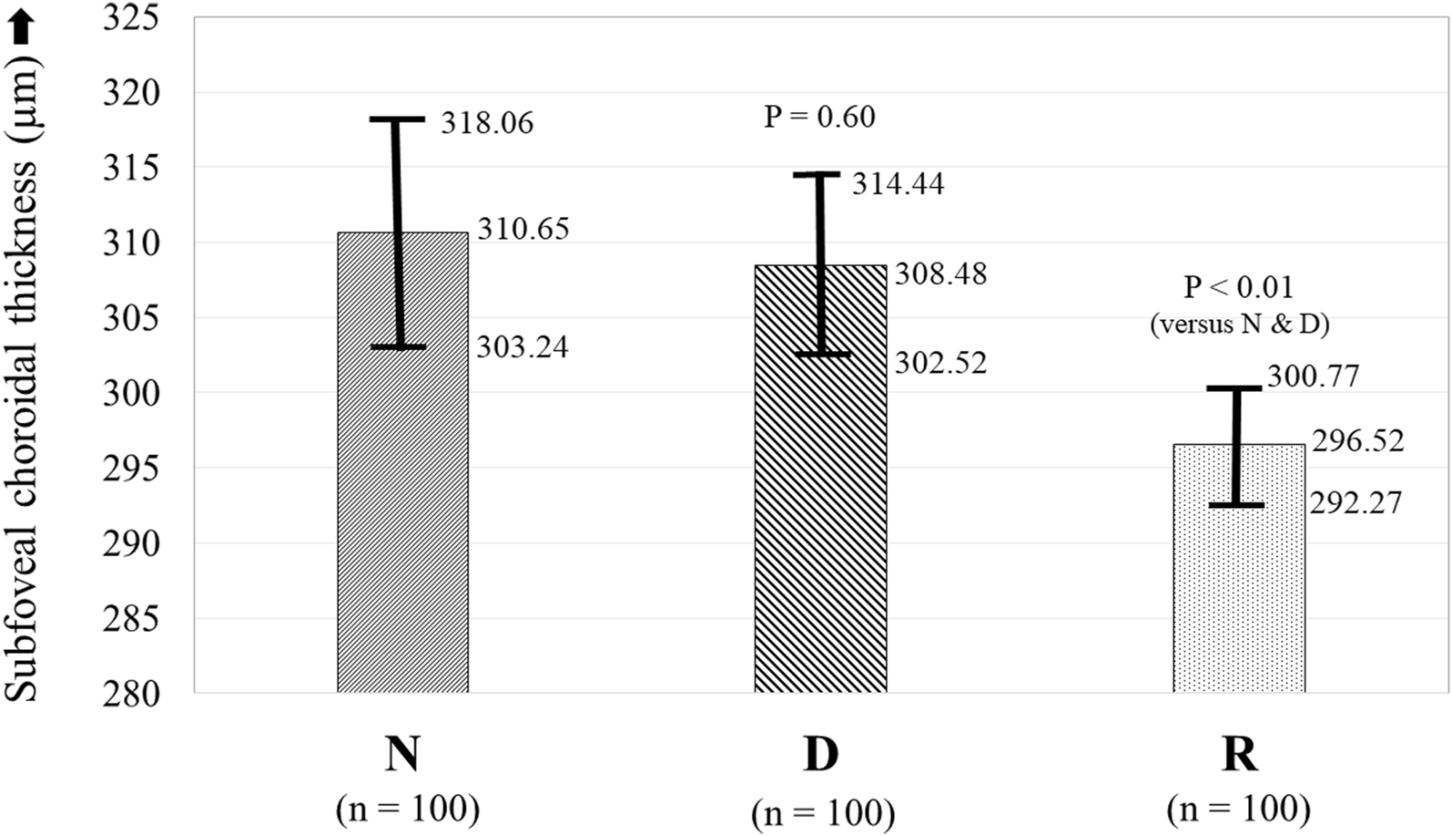
Comparison of average subfoveal choroidal thickness (with 95% confidence intervals) in eyes of non-diabetic controls (group N) versus diabetic subjects without diabetic retinopathy (group D) versus diabetic subjects with diabetic retinopathy (group R)

The baseline features of the three groups are summarised in Table 1. The baseline features in all the three groups were comparable in terms of the mean age (group N: 57.52 ± 14.30 years; group D: 59.73 ± 12.04 years; P = 0.85; group R: 62.12 ± 7.59 years; P = 0.14), sex, phakic status, prevalence of hypertension, and refractive status of the eye. The mean duration of diabetes was significantly higher in group R (14 ± 5.96 years) as compared to group D (7.47 ± 6.17 years; P < 0.01). A representative EDI-OCT image showing the choroid of a healthy subject, and that of an eye with severe NPDR, is illustrated in Fig. 1. There was no significant difference between the mean SFCT in groups N and D (N: 310.65 ± 37.34 µm; D: 308.48 ± 30.06 µm; P = 0.60) (Fig. 2). However the SFCT was significantly lower in group R (296.52 ± 21.41 µm; P < 0.01 versus both N and D) as compared to both groups N and D (Fig. 2). There was no significant difference between the SFCT in cases with type 1 DM (307 ± 16.94 µm) versus type 2 DM (308.67 ± 31.31 µm; P = 0.69).

**Table 1:** Comparison of demographics, clinical features and subfoveal choroidal thickness in healthy controls (N), diabetic patients without diabetic retinopathy (D) and diabetic patients with diabetic retinopathy (R)

	Group ‘N’ (n = 100)	Group ‘D’ (n = 100)	P value	Group ‘R’ (n = 100)	P value (vs N)	P value (vs D)
Age (years ± SD)	57.52 ± 14.30	59.73 ± 12.04	0.85	62.12 ± 7.59	0.14	0.10
Sex (M:F)	53:47	57:43	0.57	61:39	0.25	0.56
Duration of diabetes (years ± SD)	–	7.47 ± 6.17	–	14 ± 5.956	–	< 0.01*
Type of DM (1:2)	–	10:90	–	13:87	–	0.51
Hypertension (%, ratio)	24/100	30/100	0.33	26/100	0.74	0.53
Phakic: pseudophakic	85:15	71:29	0.27	81:19	0.45	0.09
Refractive status (dioptres)	− 0.28 ± 0.58	− 0.30 ± 0.68	0.82	− 0.30 ± 0.44	1.0	1.0
SFCT (µm ± SD)	310.65 ± 37.34	308.48 ± 30.06	0.60	296.52 ± 21.41	< 0.01*	< 0.01*

*N* non-diabetic, *D* diabetic without diabetic retinopathy, *R* diabetic with diabetic retinopathy, *SD* standard deviation, *M* male, *F* female, *DM* diabetes mellitus, *SFCT* subfoveal choroidal thickness

*Statistically significant

**Fig. 3:**
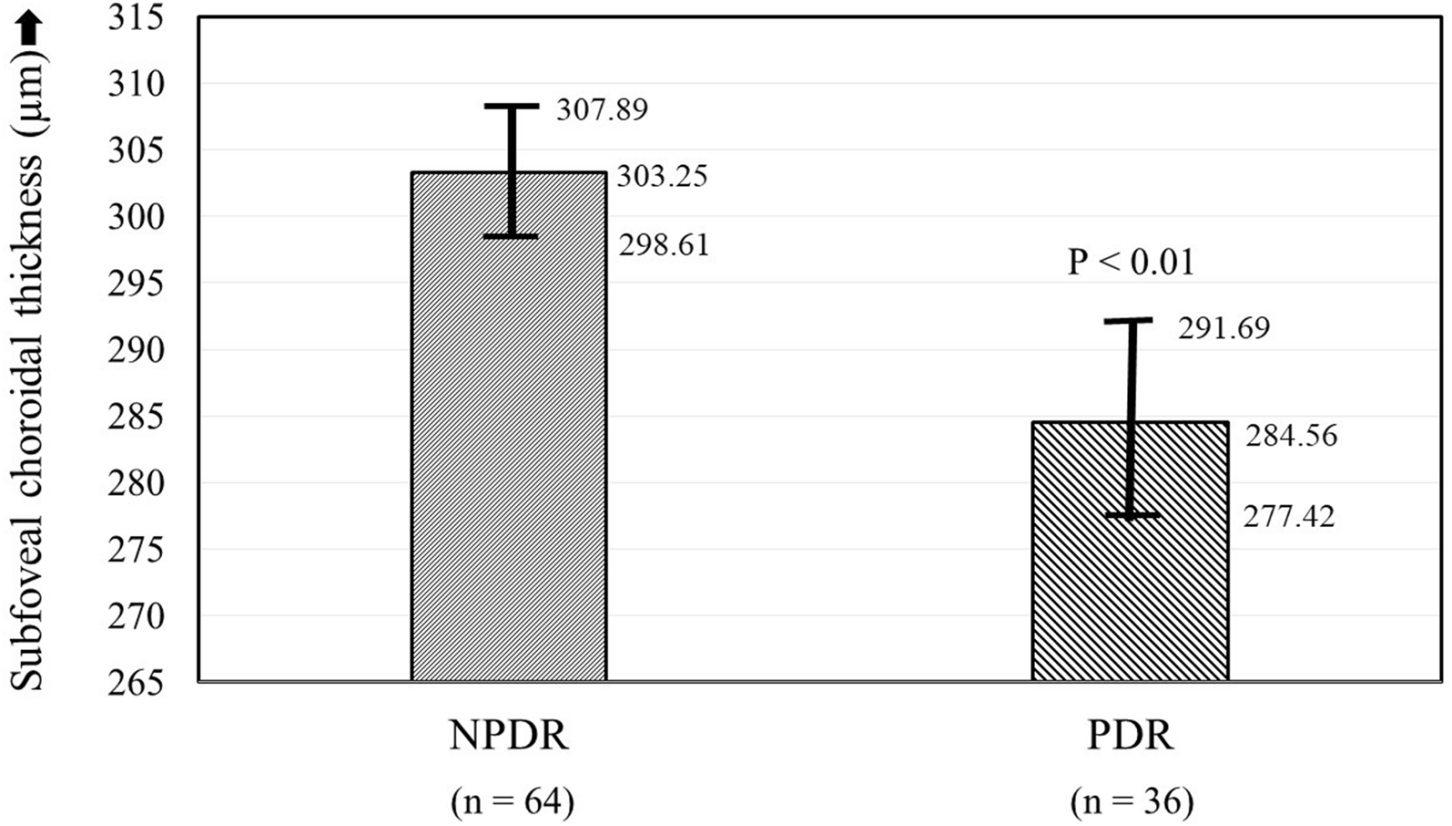
Comparison of average subfoveal choroidal thickness (with 95% confidence intervals) in eyes of diabetic subjects with non-proliferative diabetic retinopathy (NPDR) versus those with proliferative diabetic retinopathy (PDR)

On subgroup analysis of group R, 64 eyes had NPDR, while 36 eyes had PDR. Of the 64 eyes with NPDR, 16 eyes had mild (group R1), 29 eyes had moderate (group R2), and 19 eyed hade severe/very severe (group R3) stages of NPDR. All eyes with PDR (group R4, n = 36) had undergone PRP with frequency-doubled Nd:YAG laser (532 nm) more than 6 months ago. The SFCT was significantly less in eyes with PDR as compared to those with NPDR (284.56 ± 21.09 µm versus 303.25 ± 18.59 µm; P < 0.001) (Table 2, Fig. 3). However, patients with PDR had a lower mean age, higher female proportion, and a higher phakic: pseudophakic ratio as compared to patients with NPDR (Table 2). Among the eyes with DR, there was no significant difference between the SFCT in cases with type 1 DM (294.23 ± 24.66 µm) versus type 2 DM (296.86 ± 21.02 µm; P = 0.79).

**Table 2:** Comparison of demographic and clinical features, and of subfoveal choroidal thickness in diabetic patients with non-proliferative and proliferative diabetic retinopathy

	NPDR (n = 64)	PDR (n = 36)	P value
Age (years ± SD)	63.61 ± 6.85	59.47 ± 8.20	< 0.01*
Sex (M:F)	45:19	16:20	0.01*
Duration of diabetes (years ± SD)	12.80 ± 5.36	16.14 ± 6.42	< 0.01*
Type of DM (1:2)	62:2	25:11	< 0.01*
Hypertension (%, ratio)	22/64	10/36	0.50
Phakic: pseudophakic	47:17	34:2	0.01*
Refractive status (dioptres)	− 0.28 ± 0.58	− 0.30 ± 0.68	0.88
SFCT (µm ± SD)	303.25 ± 18.59	284.56 ± 21.09	< 0.01*

*NPDR* non-proliferative diabetic retinopathy, *PDR* proliferative diabetic retinopathy, *SD* standard deviation, *M* male, *F* female, *DM* diabetes mellitus, *SFCT* subfoveal choroidal thickness

*Statistically significant

**Fig. 4:**
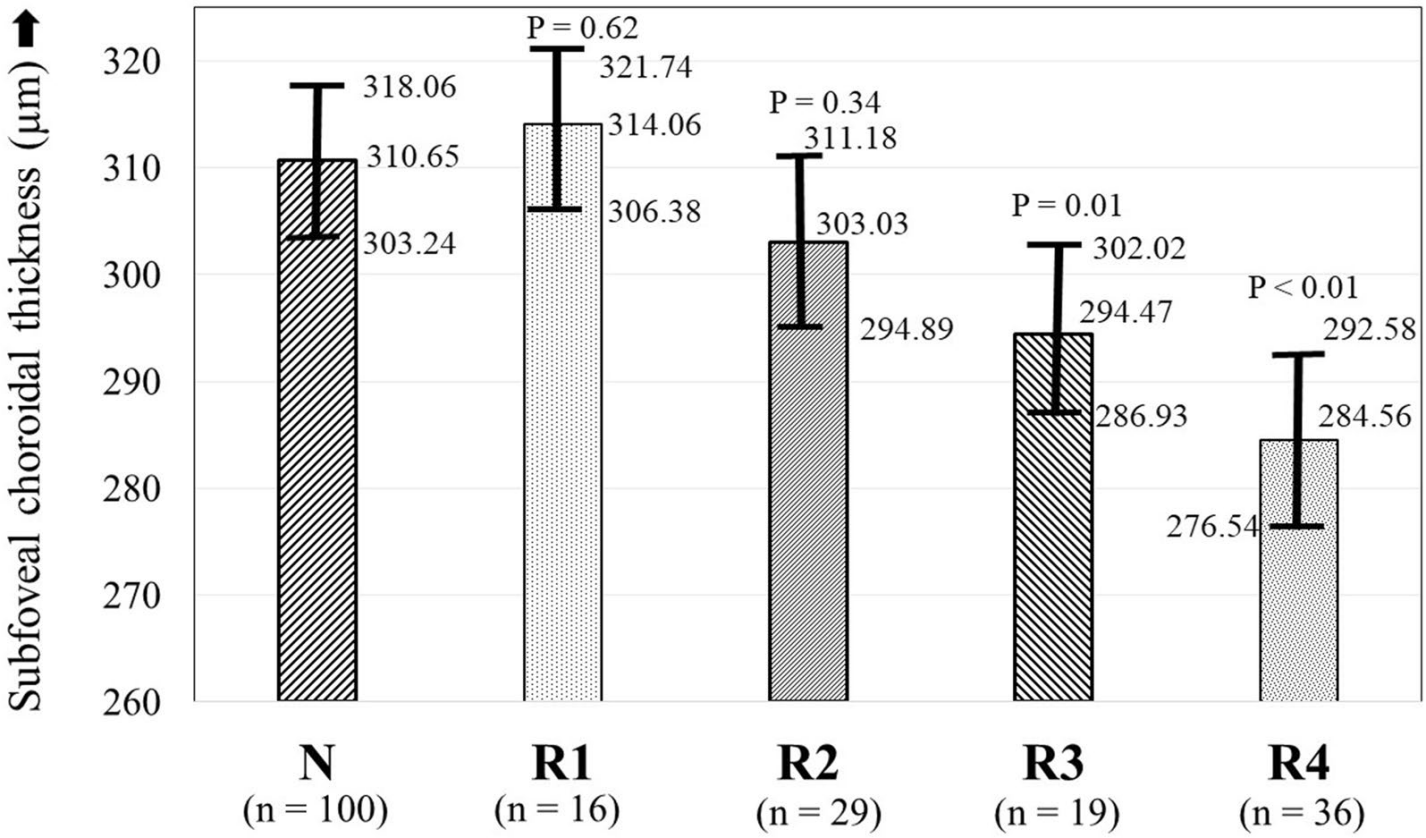
Comparison of average subfoveal choroidal thickness (with 95% confidence intervals) in normal subjects versus that in diabetic subjects with different severities of non-proliferative diabetic retinopathy (NPDR) and in proliferative diabetic retinopathy (PDR). Group R1, mild NPDR; group R2, moderate NPDR; group R3, severe NPDR; group R4, PDR; group N, normal subjects. All P-values are with respect to group N

On subgroup analysis of eyes based upon the severity of DR, there was a statistically significant moderately negative correlation of SFCT with increasing severity of DR (R = − 0.50; P < 0.01). There was a decrease in SFCT with increasing severity of DR (Fig. 4). When compared with the SFCT in group N, the change in SFCT was not significant in groups R1 (P = 0.62) and R2 (P = 0.34), but became statistically significant in groups R3 (P = 0.01) and R4 (P < 0.01). Although the difference in SFCT between groups R1 and R2 was not significant, there was a statistically significant difference in the SFCT between groups R2 and R3, and between groups R3 and R4 (R1: 314.06 ± 14.41 µm; R2: 303.03 ± 19.74 µm; P = 0.06; R3: 294.47 ± 15.65 µm; P < 0.01; R4: 284.56 ± 21.09 µm; P < 0.01) (Table 3).

**Table 3:** Comparison of demographic features and of subfoveal choroidal thickness in diabetic patients with different grades of diabetic retinopathy

	R1 (n = 16)	R2 (n = 29)	P value	R3 (n = 19)	P value (vs R1)	P value (vs R2)	R4 (n = 36)	P value (vs R1)	P value (vs R2)	P value (vs R3)
Age	62.88 ± 6.05	65.24 ± 6.98	0.27	61.74 ± 7.05	0.61	0.11	59.47 ± 8.20	0.15	< 0.01*	0.33
Sex (M:F)	0:16	4:21	0.09	8:11	< 0.01*	0.05	11:18	< 0.01*	0.07	0.77
Duration of diabetes (years ± SD)	7.75 ± 3.13	15.10 ± 4.88	< 0.01*	13.53 ± 4.86	< 0.01	0.30	16.14 ± 6.42	< 0.01*	0.51	< 0.01*
SFCT (µm ± SD)	314.06 ± 14.41	303.03 ± 19.74	0.06	294.47 ± 15.65	< 0.01*	0.03*	284.56 ± 21.09	< 0.01*	< 0.01*	0.08

*R1* mild non-proliferative diabetic retinopathy, *R2* moderate non-proliferative diabetic retinopathy, *R3* severe non-proliferative diabetic retinopathy, *R4* proliferative diabetic retinopathy, *SD* standard deviation, *SFCT* subfoveal choroidal thickness

*Statistically significant

There was a negative correlation of SFCT with the duration of diabetes (R = − 0.41; P < 0.01).

## Discussion

The current study shows that there is no significant difference between the SFCT in normal subjects and that in diabetic patients prior to the onset of DR. But there is a significant decrease in the SFCT after the onset of severe NPDR. The choroidal thinning progresses in proportion to the severity of DR.

Our findings are in agreement with Vujosevic et al. [8] who reported no significant change in the SFCT in diabetic patients without DR, and found significant choroidal thinning proportionate to the severity of DR. Sudhalkar et al. also found a significantly thinner choroid in diabetics, but in eyes both with as well as without DR [14].

Our findings are however contrary to the findings of Xu et al. [5] who reported that the subfoveal choroid in diabetic patients was significantly thicker than that in normal subjects, whereas the presence and stage of DR were not related to an abnormal SFCT.

It is known that the choroid is the only source of oxygen and nutrients to the outer retina and the retinal pigment epithelium. McLeod et al. have reported atrophy and dropout of the choriocapillaris in eyes with diabetic retinopathy [15]. We found a significant negative correlation of SFCT with the duration of diabetes. We also found that choroidal thinning was significantly present only in the diabetic cases with DR. We hypothesize that diabetic choroidopathy coexists with DR, and could be the primary event leading to some of the manifestations of diabetic retinopathy. This could explain the significantly low CT that has been reported in eyes with DR with macular ischaemia [16].

The SFCT is known to be negatively correlated with age [17, 18]. In the current study, even though the mean age of patients with PDR was significantly lower than the mean age of those with NPDR, we found that PDR is associated with a significantly thinner choroid.

The choroidal thickness has been reported to be lower in female sex [19]. The cases with PDR had a significantly higher female proportion as compared to eyes with NPDR in our study, which might also have an association with the thinner choroid in this subgroup.

There are conflicting reports on the effect of PRP on choroidal thickness in eyes with PDR. It has been reported that there is an initial increase in the SFCT after PRP [20] followed by choroidal thinning at 12 weeks [21]. However Sudhalkar et al. found no difference in the SFCT between treated (PRP done) and treatment naïve PDR. Since all cases of PDR in our study had undergone PRP more than 6 months prior to our assessment, we cannot analyse whether the choroidal thinning noted in these eyes was solely be due to diabetic choroidopathy, or was also associated with PRP.

The strength of the current study is in the overall large number of eyes enrolled in each of the major groups, as compared to most of the previous studies on the subject. However there are some inherent limitations in the current study. The measurement of SFCT may not be precise in the eyes that did not show a clear outer limit of the choroid on OCT. Another limitation is the small number of eyes in the individual subgroups with different severities of DR. Moreover we have assessed the SFCT in both eyes of each individual, which again reduces the total number of subjects, and is likely to affect the results. SFCT is a parameter that is dependent upon a number of variables and it is not feasible to have a rigorous control between the subgroups, of all those variables that are likely to intervene with the results.

## Conclusion

In conclusion, the SFCT decreases with increase in the duration of diabetes mellitus. The decrease in SFCT is proportionate to the severity of DR and becomes significant after the onset of severe NPDR.
